# The intersection of COVID-19 and cancer: signaling pathways and treatment implications

**DOI:** 10.1186/s12943-021-01363-1

**Published:** 2021-05-17

**Authors:** Zhi Zong, Yujun Wei, Jiang Ren, Long Zhang, Fangfang Zhou

**Affiliations:** 1 Institutes of Biology and Medical Sciences, Soochow University, Suzhou, 215123 China; 2 MOE Key Laboratory of Biosystems Homeostasis & Protection and Innovation Center for Cell Signaling Network, Life Sciences Institute, Zhejiang University, Hangzhou, 310058 China; 3 The Eighth Affiliated Hospital, Sun Yat-sen University, Shenzhen, 518033 China; 4 Anhui Anlong Gene Technology Co., Ltd, Hefei, 230041 China

**Keywords:** COVID-19, Cancer, Signaling pathway, Treatment implications

## Abstract

The outbreak of the novel coronavirus disease 2019 (COVID-19) caused by severe acute respiratory syndrome coronavirus 2 (SARS-CoV-2) has emerged as a serious public health concern. Patients with cancer have been disproportionately affected by this pandemic. Increasing evidence has documented that patients with malignancies are highly susceptible to severe infections and mortality from COVID-19. Recent studies have also elucidated the molecular relationship between the two diseases, which may not only help optimize cancer care during the pandemic but also expand the treatment for COVID-19. In this review, we highlight the clinical and molecular similarities between cancer and COVID-19 and summarize the four major signaling pathways at the intersection of COVID-19 and cancer, namely, cytokine, type I interferon (IFN-I), androgen receptor (AR), and immune checkpoint signaling. In addition, we discuss the advantages and disadvantages of repurposing anticancer treatment for the treatment of COVID-19.

## Background

The emergence of severe acute respiratory syndrome coronavirus 2 (SARS-CoV-2) has resulted in the novel coronavirus disease 2019 (COVID-19) pandemic. To date, more than 129 million people have been diagnosed with COVID-19, and over 2.8 million have died of COVID-19 worldwide. At the molecular level, SARS-CoV-2 infection involves the spike protein (S), which recognizes and binds to the cell surface receptor angiotensin-converting enzyme 2 (ACE2), allowing the virus to enter the host cell [1]. The process is co-opted by transmembrane serine protease 2 (TMPRSS2), a member of the serine protease family, which functions as a ‘scissor’ for S protein priming [2, 3]. Severe COVID-19, which is mostly observed in patients with comorbidities or certain medical conditions, may ultimately lead to multiple organ dysfunction and death [4, 5].

Among people in vulnerable groups, individuals with cancer were considered to be at a particularly high risk of developing adverse COVID-19 outcomes [6]. Notably, the manifestations of COVID-19 and cancer are to some extent common. For example, the unchecked overproduction of cytokines, namely the cytokine storm, is a common feature of both SARS-CoV-2 infection and cancer [7]. Furthermore, type I interferon (IFN-I) responses are indispensable for perennial immune responses against cancer and infectious diseases, and immunosuppression occurs in patients with COVID-19 and/or cancer [8]. Mechanistically, the clinical relevance of COVID-19 to cancer is based on cytokine, IFN-I, androgen receptor (AR), and immune checkpoint signaling. Understanding the underlying molecular connection between COVID-19 and cancer may help health care providers and patients reassess the risks and benefits of various therapies and make better decisions regarding suitable treatments and the timing of drug administration.

This review aims to summarize the relationship between cancer and COVID-19 from the perspective of both clinical relevance and mechanistic interplay. It emphasizes the intersecting signaling pathways between COVID-19 and cancer and discusses the opportunities and challenges of anti-SARS-CoV-2 therapies based on the molecular interplay between the two diseases.

## COVID-19 and cancer

### Susceptibility of patients with cancer to SARS-CoV-2 infection

It has long been hypothesized that patients with cancer are more vulnerable to viral infections, perhaps due to compromised immune responses [9]. The emerging COVID-19 is not an exception, as an early report revealed that patients with cancer who were treated at a tertiary cancer institution in Wuhan appeared more likely to be infected by SARS-CoV-2 [10]. Among the 1524 patients with cancer, the comparative prevalence of SARS-CoV-2 infection was twice as high as that in the overall population. In line with this, several other investigations also supported the idea that patients diagnosed with cancer have a high risk of contracting COVID-19. For example, a higher incidence of cancer was observed in COVID-19 cases (18 out of 1590) than in the general Chinese population (approximately 286 out of 100,000) [11]. A recent meta-analysis revealed that Chinese colorectal cancer patients are also more susceptible to COVID-19 infection [12]. Thus, it is now widely acknowledged that patients with cancer have a high COVID-19 risk.

**Fig. 1:**
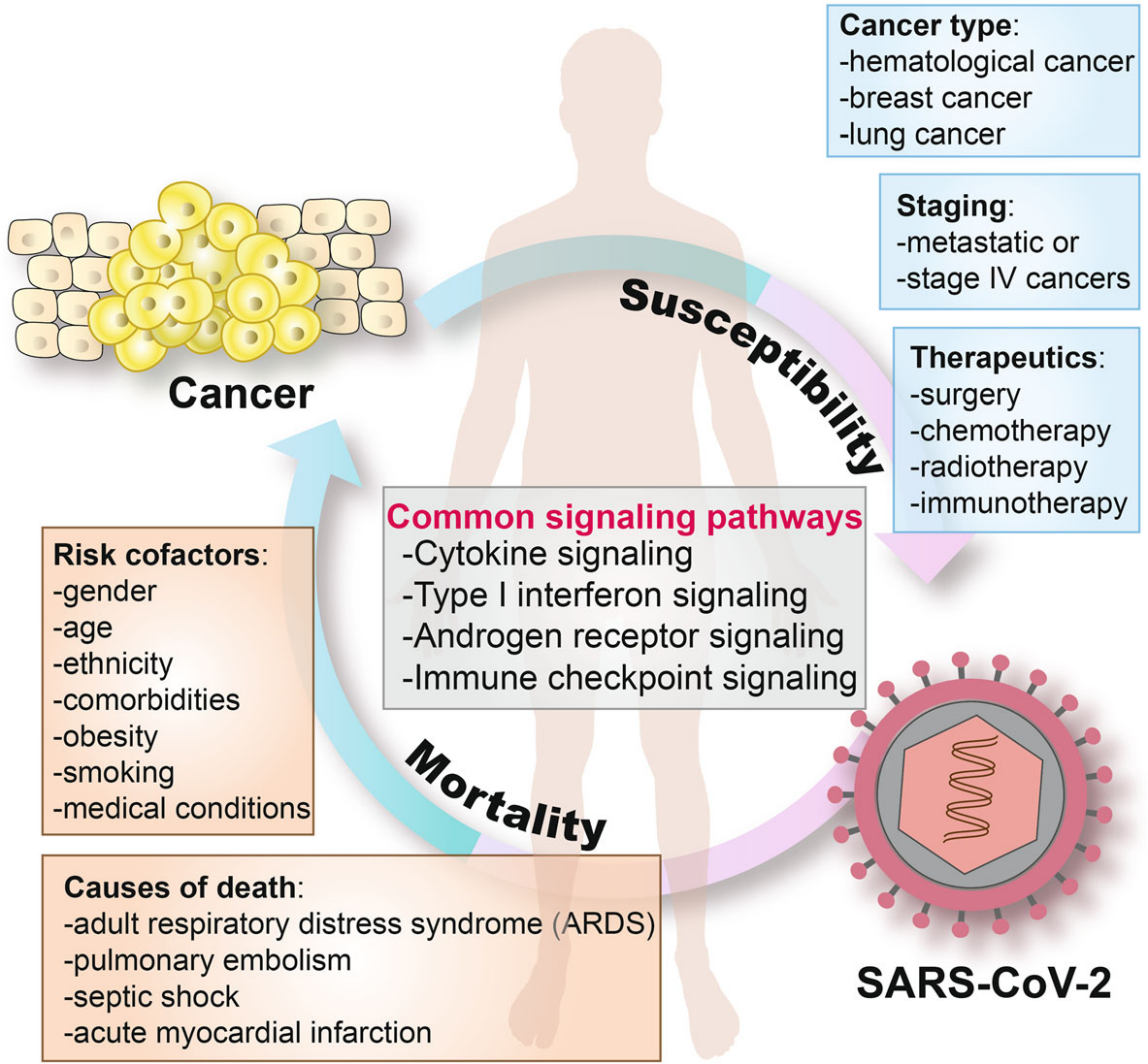
Crosstalk between coronavirus disease 2019 (COVID-19) and cancer. Patients with cancer are highly vulnerable to severe acute respiratory syndrome coronavirus 2 (SARS-CoV-2) infection. The susceptibility of patients with cancer to COVID-19 is influenced by cancer type, staging, and therapeutics. Patients with cancer who develop COVID-19 have a high risk of death. The main causes of death include adult respiratory distress syndrome (ARDS), pulmonary embolism, septic shock, and acute myocardial infarction. Other risk factors such as gender, age, ethnicity, comorbidities, obesity, smoking, and various medical conditions have been reported to have an impact on the mortality rates of patients with COVID-19. Four major signaling pathways are common to both diseases, namely cytokine, type I interferon (IFN-I), androgen receptor (AR), and immune checkpoint signaling

The susceptibility of patients with cancer to SARS-CoV-2 infection is not only reflected in the morbidity but also in the mortality, and it differs depending on the cancer type, staging, and therapeutics (Fig. 1). Different types of cancer have different effects on COVID-19 severity. For example, a study conducted in Italy reported that patients with hematological and breast cancers were more vulnerable to SARS-CoV-2 infection than those with other cancers, as breast or hematological malignancies were positively correlated with high hospitalization and mortality rates [13]. Studies have pointed out that the course of SARS-CoV-2 infection is longer, the outcome is severer, and the risk of death is much higher in patients with both lung cancer and COVID-19 than in the general US population [13, 14]. Furthermore, a study showed that patients with hematological cancer and COVID-19 have poorer outcomes than those without COVID-19 [15]. Apart from cancer type, cancer staging has also been reported to influence COVID-19 deterioration. Furthermore, severe COVID-19 symptoms were more likely to be observed in patients suffering metastatic or stage IV cancers than in those with localized malignancies [16]. Moreover, therapeutic approaches, such as surgery, chemotherapy, radiotherapy, and immunotherapy have been documented to worsen the COVID-19 outcomes among patients with cancer [8]. This is discussed in the following section.

### Mortality in COVID-19-infected patients with cancer

A number of studies have investigated the correlation between SARS-CoV-2 infection and mortality in patients with cancer. A retrospective case study in three hospitals in Wuhan revealed that among 28 patients with cancer and COVID-19, 15 patients developed severe complications, and 8 of them had died [17]. Adult respiratory distress syndrome (ARDS), pulmonary embolism, septic shock, and acute myocardial infarction were the leading causes of death [18]. Similar high mortality rates were also reported in patients with hematological cancer with concurrent COVID-19 in China and in the first 25 patients with cancer in Italy [18, 19]. Moreover, a population-based study conducted in Italy showed that among the patients diagnosed with COVID-19, 9.5% of men had a cancer diagnosis. Intriguingly, among patients with COVID-19, men with cancer were more likely to die compared to men without cancer [20]. Multivariate mortality analyses in COVID-19 were recently conducted by comparing 312 patients with cancer and 4833 patients without cancer in Louisiana, USA. The large study showed that patients with cancer who are over 65 years old, those with certain comorbidities, and those who received cancer-directed therapy harbor the greatest risk of death, further supporting the hypothesis that patients with cancer are at increased risk for mortality [21]. Conversely, a large group of SARS-CoV-2-infected patients who developed cancer in New York City showed no significant differences in mortality compared to non-cancer patients [22]. A possible explanation for the controversies is that a number of cofactors have not been taken into account in assessing the mortality of COVID-19 in patients with cancer. For example, gender, age, ethnicity, comorbidities (including cardiovascular and respiratory), obesity, smoking, and various medical conditions have been reported to have a tremendous impact on the mortality rates of patients with COVID-19 [15, 23–29] (Fig. 1). Other variables such as socio-economic factors were not included in the study. A worldwide comparison showed correlations between COVID-19 cases and gross domestic product (GDP) [30]. A number of studies indicated that communities with a high African American density have been disproportionately burdened with COVID-19, although it is unclear whether there are more COVID-19 cases in patients with cancer in the African American community [31–33]. Nevertheless, these findings suggest that socio-economic factors are also vital variates that may affect the association between cancer and COVID-19. Thus, more factors should be considered in future analyses, and the effects of each factor should be investigated separately to determine the probabilities of death in patients with cancer and COVID-19.

## Common signaling pathways in COVID-19 and cancer

### Cytokine signaling

During the early stage of the pandemic, the upregulation of many cytokines, including interleukin (IL)-6, IL-1β, tumor necrosis factor α (TNF-α), and interferons, was observed in patients with COVID-19 [34, 35]. Elevated cytokine levels may cause a putative systemic outcome, known as a cytokine storm or cytokine release syndrome (CRS). CRS results from an excessive immune response and is believed to cause a substantial increase in proinflammatory cytokines in response to immune system diseases and/or neoplasia [36]. The increase in cytokines may lead to widespread tissue damage, such as acute lung injury. ARDS, as a more severe form of acute lung injury, causes a lower oxygen saturation level, thus leading to multi-organ failure. Regarding ARDS caused by COVID-19, a study demonstrated that nearly half of the patients with COVID-19 progressed to ARDS [37]. Given the serious consequences, ARDS is believed to be the major cause of mortality in COVID-19 [38]. Although over 50 cytokines and growth factors have been implicated in this abnormal signaling response, the proinflammatory cytokine IL-6, with biological functions in immunity, tissue regeneration, and metabolism, plays a major role in this process [39]. Importantly, IL-6 was found to be elevated in the serum of patients with COVID-19 [40]. Given that IL-6 was also reported to be aberrantly hyperactivated in many types of cancer [41], in this section, we will discuss the interplay of COVID-19 and cancer in IL-6/Janus kinase (JAK)/signal transducer and activator of transcription (STAT) signaling.

#### IL-6 and JAK/STAT signaling

**Fig. 2:**
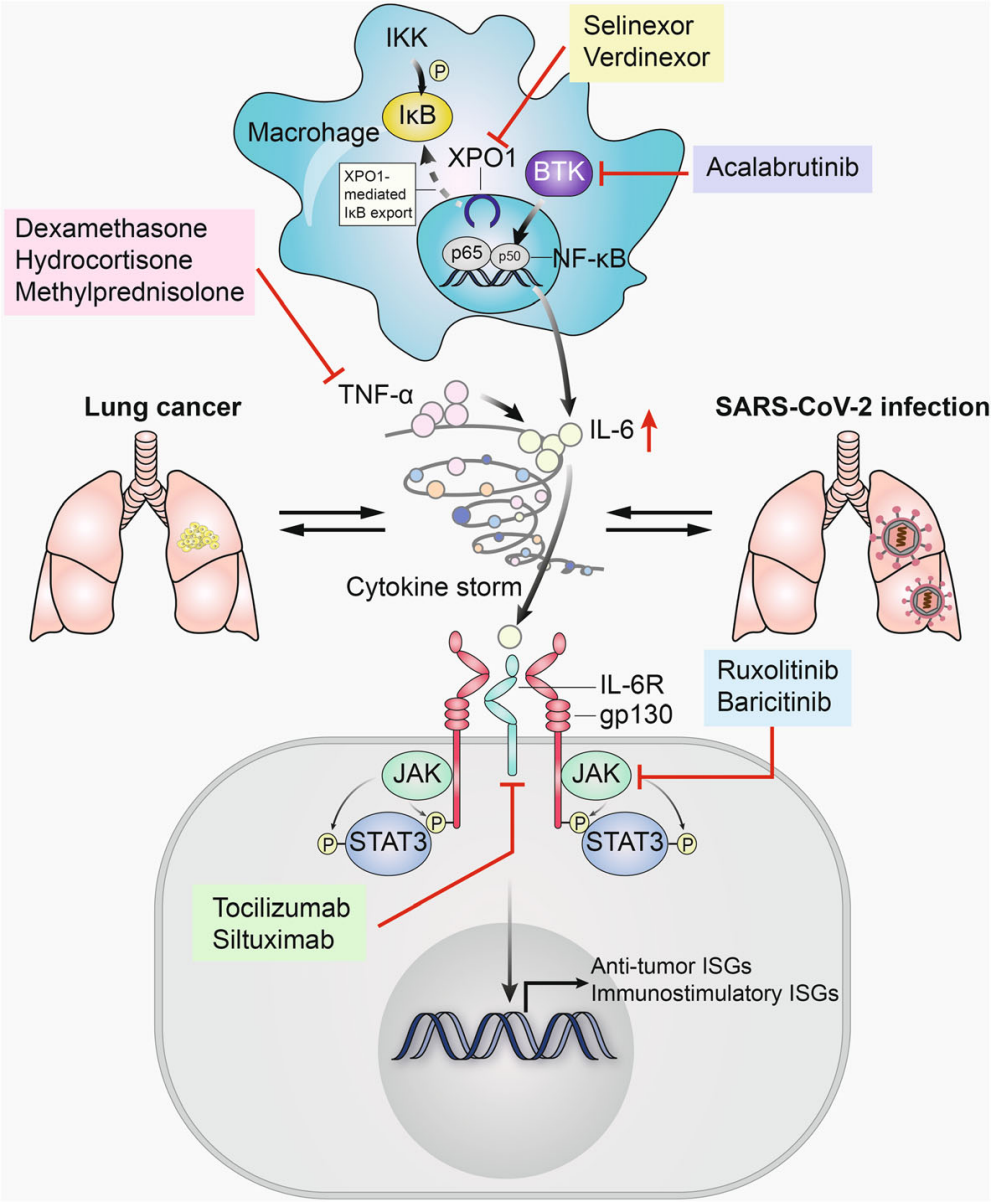
Cytokine signaling in lung cancer and coronavirus disease 2019 (COVID-19). Interleukin (IL)-6 accumulates as a result of nuclear factor (NF)-κB activation or stimulation by other cytokines, such as tumor necrosis factor (TNF)-α. The accumulated IL-6 plays a key role in a systemic hyperactivated immune response known as the cytokine storm. IL-6 serves as a driver of tumor progression, and several cancer-related risk factors may in turn boost IL-6 expression, driving the unfavorable cytokine storm. Furthermore, cytokine overproduction exacerbates COVID-19, and elevated IL-6 levels have been observed in patients with severe COVID-19. IL-6-mediated Janus kinase (JAK)/signal transducer and activator of transcription (STAT) signaling is initiated by the binding of IL-6 to the IL-6 receptor (IL-6R) and subsequent interaction with gp130. The IL-6/IL-6R/gp130 complex, then, activates JAK enzymes, which phosphorylate gp130, providing docking sites for STAT3. JAK, then, phosphorylates STAT3 and subsequently induces the transcription of multiple target genes. XPO1 inhibitors such as selinexor and verdinexor abolish XPO1-mediated IκB export, thus reducing NF-κB pathway and decreasing the production of proinflammatory cytokines. Bruton tyrosine kinase (BTK) inhibitors inhibit NF-κB signaling, resulting in reduced IL-6 production. Corticosteroids inhibit TNF-α-mediated IL-6 mRNA expression. Other targeted therapeutics for the treatment of cancer and COVID-19, such as IL-6R antibodies and JAK inhibitors, are shown

JAK/STAT signaling plays a pivotal role in regulating cell growth, survival, differentiation, motility, and immune responses. This pathway mediates the effects of a large number of cytokines and growth factors. Aberrant hyperactivation of the JAK/STAT pathway may result in chronic inflammatory conditions or various types of cancer [42]. IL-6-mediated JAK/STAT signaling consists of three distinct pathways, among which the classic and *trans*-signaling pathways are the most well studied. The classic signaling pathway is initiated by the binding of IL-6 to the IL-6 receptor (IL-6R) on the cell membrane and subsequent interaction with the transmembrane protein IL-6 receptor subunit-beta (gp130, also known as IL-6Rβ). In contrast, the *trans*-signaling pathway involves the binding of IL-6 to a soluble form of IL-6R (sIL-6R), followed by the formation of a complex between IL-6-sIL-6R and gp300 [43]. Another mode of IL-6 signaling, known as IL-6 trans-presentation, has recently been identified and involves specialized dendritic cells [44]. The engagement of gp130 results in the activation of JAK enzymes, which subsequently phosphorylate several tyrosine residues on gp130, providing docking sites for proteins, such as STAT3, initiating downstream signaling. Once STAT3 binds to phosphorylated gp130, JAK phosphorylates STAT3, leading to STAT3 homo-dimerization. Subsequently, the STAT3 dimer translocates into the nucleus, where it induces the transcription of multiple target genes [42] (Fig. 2).

#### IL-6-mediated cytokine storms in cancer

IL-6 triggers the production of excess proinflammatory cytokines in the tumor microenvironment. This chronic inflammatory environment subsequently causes carcinogenesis.

It has been reported that IL-6 serves as a driver of tumor progression, as well as a biomarker of cancer diagnosis and prognosis [45]. Elevated IL-6 levels have been reported in patients with various types of cancer, such as breast, pancreatic, colorectal, prostate, and non-small cell lung cancer (NSCLC) [46–50]. Moreover, preclinical studies have demonstrated that IL-6 is associated with the severity of various cancer types, such as breast cancer and pancreatic cancer [51–53]. In breast cancer, IL-6 has been shown to promote tumor stem cell self-renewal and epithelial-to-mesenchymal transition (EMT), thus facilitating breast cancer metastasis [51, 54, 55]. Notably, IL-6 levels are also diagnostic markers of therapeutic response and prognostic indicators of survival probability in certain types of cancer [45, 50, 56–60].

Several factors may contribute to IL-6-induced neoplastic changes. For example, exposure to carcinogens, such as cigarette smoking, has been shown to induce mutations in *KRAS*, which in turn boost IL-6 expression levels in the lung epithelium, facilitating lung adenocarcinoma pathogenesis through the JAK/STAT3 pathway [61, 62]. In addition to carcinogen exposure, chimeric antigen receptor (CAR) T cell therapy has been shown to generate IL-6, which drives the undesired “cytokine storm” in the treatment of chemorefractory hematological malignancies and some solid tumors [63]. Redundant amounts of IL-6 are also produced by tumor-associated exhausted CD8+ T lymphocytes [64]. These findings indicate that the IL-6/JAK/STAT signaling pathway plays a crucial role in cancer biology.

#### IL-6 in COVID-19

Cytokine responses have been proposed as the cause of severe coronavirus infection in humans [65]. As the COVID-19 pandemic continues to rage, recent advances in the pathogenesis of SARS-CoV-2 infection have again put a cytokine storm on the stage and suggest its direct correlation with lung injury, multi-organ failure, and adverse prognosis of severe COVID-19 [4, 34, 66–68]. Immune dysregulation in patients with severe COVID-19 is driven by either overproduction of proinflammatory cytokines downstream of IL-6 or CD4 lymphopenia-induced lymphocytic dysregulation [69]. Increased levels of proinflammatory cytokines, including IL-2, IL-6, IL-7, IL-10, IFNγ, and TNF-α, are commonly observed in patients with severe COVID-19 [34, 70, 71]. Moreover, the expression levels of cytokines are closely correlated with the viral load and lung injury, reflecting the severity and prognosis of this disease [72, 73]. According to the results of another study, the increase in IL-6 was regarded to reflect the shift from tissue-resident alveolar macrophages to IL-6-producing monocyte-recruited macrophages, as observed in bronchoalveolar lavage (BAL) samples from patients with severe COVID-19 compared to those from patients with moderate COVID-19 [74]. Recently, a retrospective cohort study comparing the immunological characteristics between 93 COVID-19 patients with cancer and 1959 COVID-19 patients without cancer. COVID-19 patients with cancer were reported to have significantly elevated inflammatory cytokines, as well as decreased immune cells than those without cancer [75]. These observations revealed that the immunological alternation, especially cytokine storm, is a key indicator of COVID-19 deterioration.

#### Targeting IL-6/JAK/STAT signaling

Given that overproduction of plasma IL-6 levels was observed in patients with severe COVID-19, and also in patients with disseminated malignancies, it is conceivable that IL-6, or its downstream molecules, could be a promising target for the treatment of COVID-19. Therefore, drugs targeting the IL-6/JAK/STAT pathway with anticancer effects may be repurposed for the treatment of COVID-19, saving invaluable time and allowing timely delivery of care.

Nuclear factor-κB (NF-κB) signaling plays a key role in the production of a number of chemokines and cytokines and is activated by viral genetic materials or proteins [76, 77]. The trafficking of the key protein IκB between the nucleus and cytoplasm is indispensable for proper functioning. In this process, exportin 1 (XPO1, also known as CRM1) is responsible for the export of proteins from the nucleus. Studies have shown that XPO1 contributes to host immunopathology during viral infection [78]. Based on these observations, it is conceivable that blocking XPO1 may contribute to the suppression of the NF-κB pathway and reduction in cytokine production. In cancer biology, XPO1 is highly expressed and overactivated in many cancers, causing the improper localization and consequent dysfunction of important tumor suppressors [79]. Drugs in selective inhibitors of nuclear export (SINE) family, such as selinexor and verdinexor, showed effectiveness in blocking XPO1 and maintaining the proper localization of anti-tumor proteins [80]. Selinexor has shown promising anti-tumor activity in patients with hematological malignancies and has received US Food and Drug Administration (FDA) approval for the treatment of penta-refractory multiple myeloma [81–84]. Besides, a number of clinical trials with selinexor are evaluating its efficacies in treating solid tumors. In terms of inhibiting NF-κB pathway, KPT-350, another SINE compound, has been shown to upregulate anti-inflammatory cytokines (such as IL-4, IL-10, and IL-13), as well as downregulate proinflammatory cytokines (such as IL-6) [85]. SINE compounds have also been shown to exhibit anti-inflammation effects against ARDS, which is similar to that seen in SARS-CoV-2 [86]. In influenza virus-infected mice, verdinexor was shown to lower the expression of proinflammatory cytokines and reduce inflammation [87]. Importantly, besides the anti-inflammation effects, XPO1-mediated protein or RNA export is also necessary for viral replication [88, 89]. In view of these facts, clinical trials have been initiated to evaluate the safety and effectiveness of XPO1 inhibitors in patients with COVID-19 (Table 1).

**Table 1:** Potential drugs with cancer indications for treatment of COVID-19

Drugs	Targets	Cancer indication	Antiviral indication	Clinical trial identifier
Anti-cytokine therapies				
SINE compounds (Selinexor; Verdinexor)	XPO1	Multiple myeloma [90–92], non-Hodgkin lymphoma [92], acute myeloid leukemia [93, 94], solid tumors [84, 95]	Influenza viruses [87]	NCT04349098, NCT04349098, NCT04355676
Acalabrutinib	BTK	Specific B cell malignancies [96, 97]	COVID-19 [98]	NCT04394884, NCT04380688, NCT04346199, NCT04647669
Corticosteroids (Dexamethasone; Hydrocortisone; Methylprednisolone)	TNF-α	Hematological malignancies [99]	COVID-19 [100, 101]	NCT04648410, NCT04654416, NCT04359511, NCT04530409, NCT04586114, NCT04451174, NCT04484493, NCT04344288
Tocilizumab	IL-6R	Various cancers, such as pancreatic cancer, ovarian cancer, and colitis-associated colorectal cancer [102–104]	COVID-19 [105–107]	NCT04320615, NCT04372186, NCT04370834
Siltuximab		Various cancers, such as ovarian cancer, lung cancer [108, 109]	COVID-19 [110]	NCT04486521, NCT04330638, NCT04329650
Ruxolitinib	JAK1/2	myeloproliferative neoplasms [111]	COVID-19 [112, 113]	NCT0435579, NCT04362137, NCT04377620, NCT04334044, NCT04337359, NCT04338958, NCT04348695, NCT04354714
Baricitinib		Non-melanoma skin cancer [114]	COVID-19 [115, 116]	NCT04358614, NCT04340232, NCT04373044, NCT04393051, NCT04320277, NCT04399798, NCT04346147, NCT04362943
Interferon-based therapies				
IFNα or IFNβ	N/A	Hematological cancers [117]	Hepatitis B and C HIV [118] COVID-19 [119–121]	NCT04344600, NCT04350671, NCT04343768. NCT04343976, NCT04254874, NCT04320238, ChiCTR2000029387, NCT04315948, NCT04276688
Androgen-deprivation therapies				
Enzalutamide	Androgen receptor (AR)	Prostate cancer [122]	COVID-19 [3]	NCT04475601
Apalutamide				N/A
Darolutamide				N/A
Proxalutamide				NCT04446429, NCT04728802
Bicalutamide				NCT04509999
Camostat	TMPRSS2			NCT04652765
Nafamostat				NCT04418128, NCT04390594, NCT04352400, NCT04628143, NCT04623021, NCT04473053
Bromhexine				NCT04355026, NCT04405999, NCT04424134
Immune checkpoint inhibitors				
Pembrolizumab	PD-1	Various cancers [123–125]	HIV [126] HBV/HCV [127, 128] COVID-19 [129]	NCT04335305
Nivolumab				NCT04413838, NCT04356508, NCT04343144
Monalizumab	NKG2A	Various cancers such as ovarian cancer, squamous cervical cancer, and epithelial endometrial cancer [130]	COVID-19 [131]	NCT04333914
Avdoralimab	C5aR	Solid tumors such as cervical cancer and breast cancer [132, 133]	COVID-19 [134]	NCT04333914

Upon viral infection, Bruton tyrosine kinase (BTK), which is downstream of toll-like receptors (TLRs), is activated and initiates NF-κB signaling. Accordingly, BTK inhibitors may regulate inflammatory responses in COVID-19 by reducing the levels of IL-6. BTK inhibitors, such as acalabrutinib, have already been applied in the treatment of chronic lymphocytic leukemia [135]. Acalabrutinib was administered to 19 patients with severe COVID-19 to evaluate its efficacy in regulating inflammatory responses [98]. As expected, most patients manifested reduced levels of IL-6 and C-reactive protein (CRP), as well as improved oxygenation. Clinical trials are under evaluation to gain insights into the role of BTK inhibition in improving COVID-19 by reducing the levels and effects of IL-6.

Apart from BTK inhibitors, corticosteroids have been proven to exhibit anti-IL-6 activities. Corticosteroids have been used as cytotoxic agents for treating hematological cancers, such as acute lymphoblastic leukemia (ALL), by inhibiting lymphoid development. A clinical trial conducted in the UK showed that after dexamethasone treatment, decreased mortality rates were observed in patients developing COVID-19 [100]. Mechanistically, dexamethasone destabilizes IL-6 mRNA and inhibits TNF-α-mediated IL-6 mRNA expression and subsequent protein secretion. Given the availability and inexpensiveness of steroids, several randomized controlled trials have been conducted by the WHO to assess the efficacies of various steroids, including dexamethasone, hydrocortisone, and methylprednisolone [101]. The results of these trials are satisfactory, as patients receiving corticosteroids show a decreased possibility of death compared to those receiving standard care or placebo. Notably, recently a meta-analysis of randomized clinical trials was conducted to evaluate the effect of corticosteroid therapy in patients with different disease severity. In this study, the researchers reckoned that corticosteroids may be considered in patients with critical COVID-19 rather than those not requiring oxygen therapy, as these two subgroups showed a significantly different effect on survival [136].

Tocilizumab, an antibody targeting human IL-6R, disrupts both the classic and *trans*-signaling pathways. Previous findings have demonstrated the validity of tocilizumab against many types of cancer, such as pancreatic, ovarian, and colitis-associated colorectal cancers [102–104]. Strikingly, tocilizumab has received approval from the Chinese government for the treatment of pulmonary complications related to severe COVID-19. Tocilizumab has been proven to be effective in several basic and clinical studies. For example, in an observational study, 21 Chinese patients with critical COVID-19 exhibited an improvement in both clinical and radiological outcomes after tocilizumab administration [105]. Additionally, tocilizumab was proven successful in treating COVID-19-related respiratory failure, as well as multiple myeloma in a COVID-19 patient [106, 107]. From a molecular perspective, tocilizumab blocks IL-6R and, thus, attenuates aberrantly activated immune responses [137]. Furthermore, it restores adaptive immunity by rejuvenating T cells [138].

Siltuximab, an alternative monoclonal antibody targeting IL-6R, has been widely studied in cancer. Preclinical studies have shown that siltuximab exerts antitumor activities accompanied by reduced levels of activated STAT3 and MAPK in some solid tumors. With regard to treating COVID-19, an observational study revealed the improvement of outcomes in most patients after receiving siltuximab, as demonstrated by a decrease in IL-6 and CRP levels [110].

Several commercially available drugs target JAKs. Ruxolitinib, a small-molecule tyrosine kinase inhibitor of JAK1 and JAK2, may decrease lymphocyte activation and reduce proinflammatory cytokine secretion. It was approved by the FDA for treating myeloproliferative neoplasms as well as polycythemia vera [111, 139, 140]. Baricitinib, another JAK1/2 inhibitor, was previously used to treat rheumatoid arthritis [141]. In addition, a clinical trial is evaluating the efficacy of baricitinib in the prevention of graft-versus-host disease (GVHD) in patients with hematological malignancies after peripheral blood donor stem cell transplantation. Importantly, a study identified baricitinib as a potential treatment for COVID-19 [115]. Mechanistically, baricitinib alleviates viral infection by inhibiting AP2-associated protein kinase 1 (AAK1), a known regulator of endocytosis. It is believed that AAK1 disruption may interfere with the passage of the virus into cells as well as the intracellular assembly of virus particles [142]. Considering its role in JAK/STAT signaling, a number of clinical trials aimed at treating COVID-19 are under evaluation (Table 1).

### IFN-I signaling

**Fig. 3:**
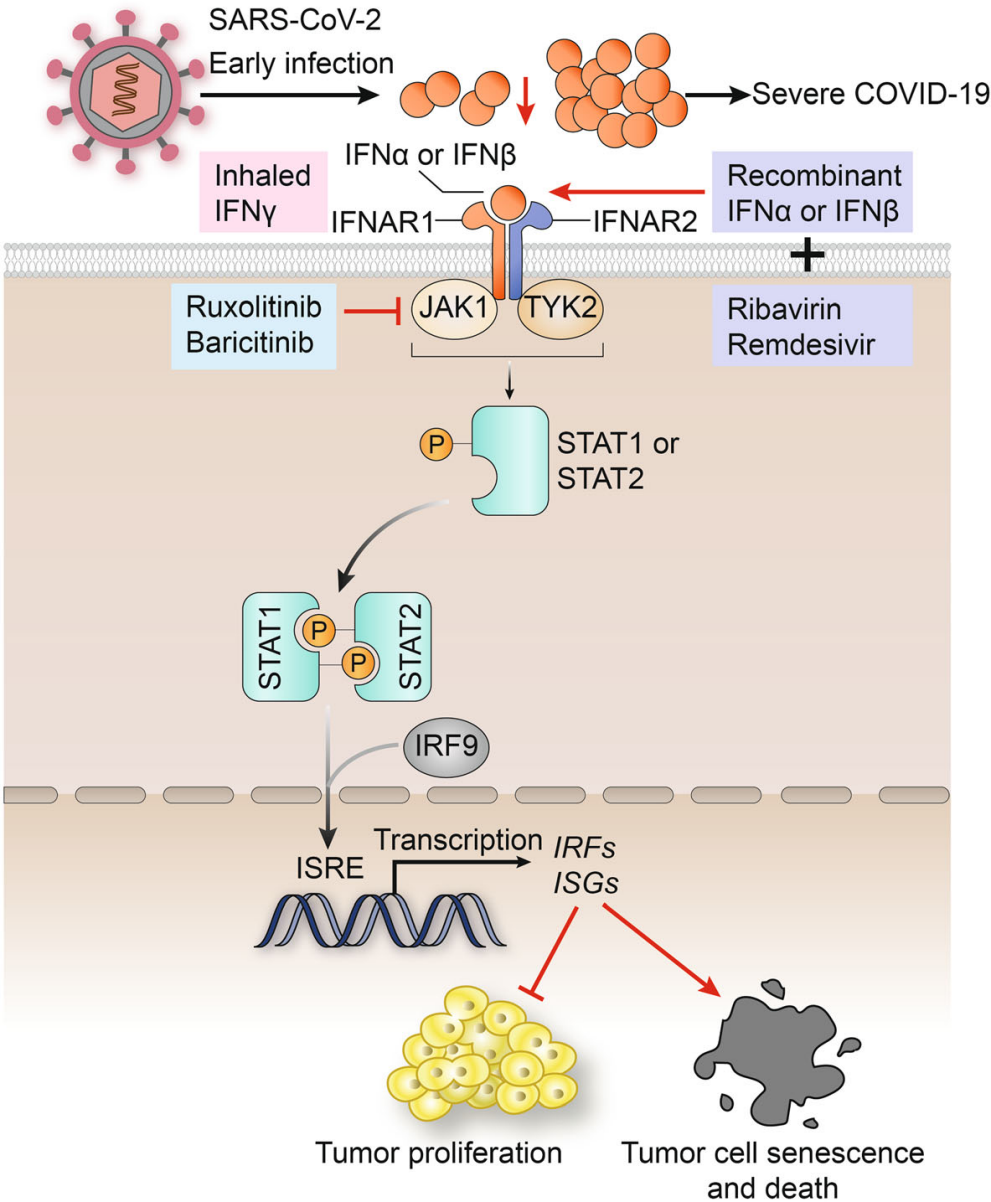
Type I interferon (IFN-I) signaling in cancer and coronavirus disease 2019 (COVID-19). IFN-I plays a key role in inhibiting tumor proliferation and promoting tumor cell senescence and death, whereas impaired IFN-I signaling is associated with tumor progression. In early severe acute respiratory syndrome coronavirus 2 (SARS-CoV-2) infection, IFN-I signaling is dampened, while IFN-I responses may aggravate unfavorable inflammation and the progression of severe COVID-19. IFN-Is include IFNα and IFNβ, which signal through binding to the common receptor IFNR (homodimer of IFNAR1 and IFNAR2). Then, JAKs are activated to initiate signal transduction via STATs. STATs, along with IRF9, enter the nucleus, leading to the activation of a multitude of interferon regulatory factors (IRFs) and IFN-stimulated genes (ISGs). IFN-I-based therapies for cancer and COVID-19 are shown

IFNs belong to a large family of cytokines, which are currently classified into three groups (type I, II, and III IFNs), according to their receptor specificity and sequence homology. Type I IFNs comprise a single IFNβ gene and 13 IFNα genes in humans, and signal through a common receptor, IFNR, which is formed by the heterodimerization of IFNAR1 and IFNAR2. Akin to IL-6, IFN-Is bind to the receptor activating JAKs to initiate signal transduction through the JAK/STAT pathway. Consequently, IFN-I signaling leads to the activation of a multitude of interferon regulatory factors (IRFs) and IFN-stimulated genes (ISGs), thus promoting inflammatory and innate antiviral responses [143] (Fig. 3).

#### IFN-I signaling in cancer and COVID-19

As said, IFN-I responses are indispensable for immune responses against both cancer and infectious diseases [144–146]. In cancer biology, IFN-I plays a vital role in inhibiting tumor proliferation and promoting tumor cell senescence and death, and impaired IFN-I signaling is associated with tumor progression [147, 148]. Although the underlying mechanism of the inhibitory effect of IFN on cancer cells is limited, early studies have shown that the combined process of cell cycle arrest and cell death may explain the inhibition of tumor cell expansion mediated by IFN-Is [149, 150].

Frequent studies have demonstrated the positive role of IFN-I responses during the early stage of viral infection. Like many other viruses, SARS-CoV-2 also evolved mechanisms to evade host antiviral responses. In support of this, an early study showed that IFN-I signaling was dampened in response to SARS-CoV-2 infection [151]. Moreover, low levels of IFN-I and ISGs, along with an increase in IL-6 and inflammatory responses, were observed in peripheral blood samples from patients with severe or critical COVID-19 [152].

However, contradictory results have been reported regarding IFN-I responses in patients with COVID-19. Several studies have shown that patients with COVID-19 exhibit robust IFN-I responses, illustrated by increased expression of ISGs in the bronchoalveolar lavage fluid or peripheral blood mononuclear cells (PBMCs) [153, 154]. More recently, single-cell RNA sequencing analysis has shown that IFN-I responses co-occur with TNF- and IL-1-driven inflammatory responses in PBMCs from patients with severe COVID-19, rather than mild COVID-19 [155]. This suggests the role of IFN-I responses in aggravating unfavorable inflammation and the progression of severe COVID-19. In addition, a longitudinal analysis demonstrated that in severe COVID-19, IFNα in peripheral blood is expressed at high levels in a continuous manner [156]. The excessive inflammatory responses of IFN-I have also been described in separate mouse models. For example, improper timing of the IFN-I response failed to inhibit viral replication of MERS-CoV and SARS-CoV-2 [157, 158]. During SARS-CoV infection, a delayed but considerable IFN-I response increases infiltration and recruitment of monocytes and macrophages to the infected lungs, resulting in fatal pneumonia and depleted T cell responses [159].

#### IFN-I-based therapies

Considering the opposing reported results for the robustness of IFN-I responses at different stages of disease progression, more investigations should be conducted to determine the appropriate timing of IFN-I activation for antiviral responses. Nevertheless, due to the extensive antiviral activities of IFN-I, IFN-I-based therapy has shown great benefits in both chronic viral infection and cancer, as reviewed elsewhere [118, 144]. Recombinant IFN-Is, such as IFNα and IFNβ, are now being actively studied as a therapeutic approach for COVID-19. In the clinic, IFN-Is, either alone or in combination with other antiviral agents, such as ribavirin or remdesivir, are currently being tested for their clinical efficacy against COVID-19 [119–121]. Given the potential side effects of hyperinflammation in the severity of COVID-19, the timing of IFN-I administration requires careful consideration. A retrospective multicenter cohort study reported that early interferon therapy was associated with a high probability of survival, whereas delayed administration led to the opposite outcome [160]. Thus, it will be necessary to examine SARS-CoV-2 viral loads, virulence, and the expression of IFN-I-related genes to estimate the suitable time for IFN therapy against COVID-19. An emerging approach to increase the systemic circulating levels of IFN-Is would be to use IFN-III (IFNγ) as an inhaled aerosol, as it has been proven to be safe and effective in improving pulmonary function in patients with idiopathic pulmonary fibrosis [161].

In the context of repressing proinflammatory IFN-I responses, the therapeutic strategy is similar to that of the IL-6-mediated cytokine storm. Therefore, the administration of JAK inhibitors should be considered for the treatment of patients with severe COVID-19 to dampen overactivated IFN-I signaling.

### AR signaling

**Fig. 4:**
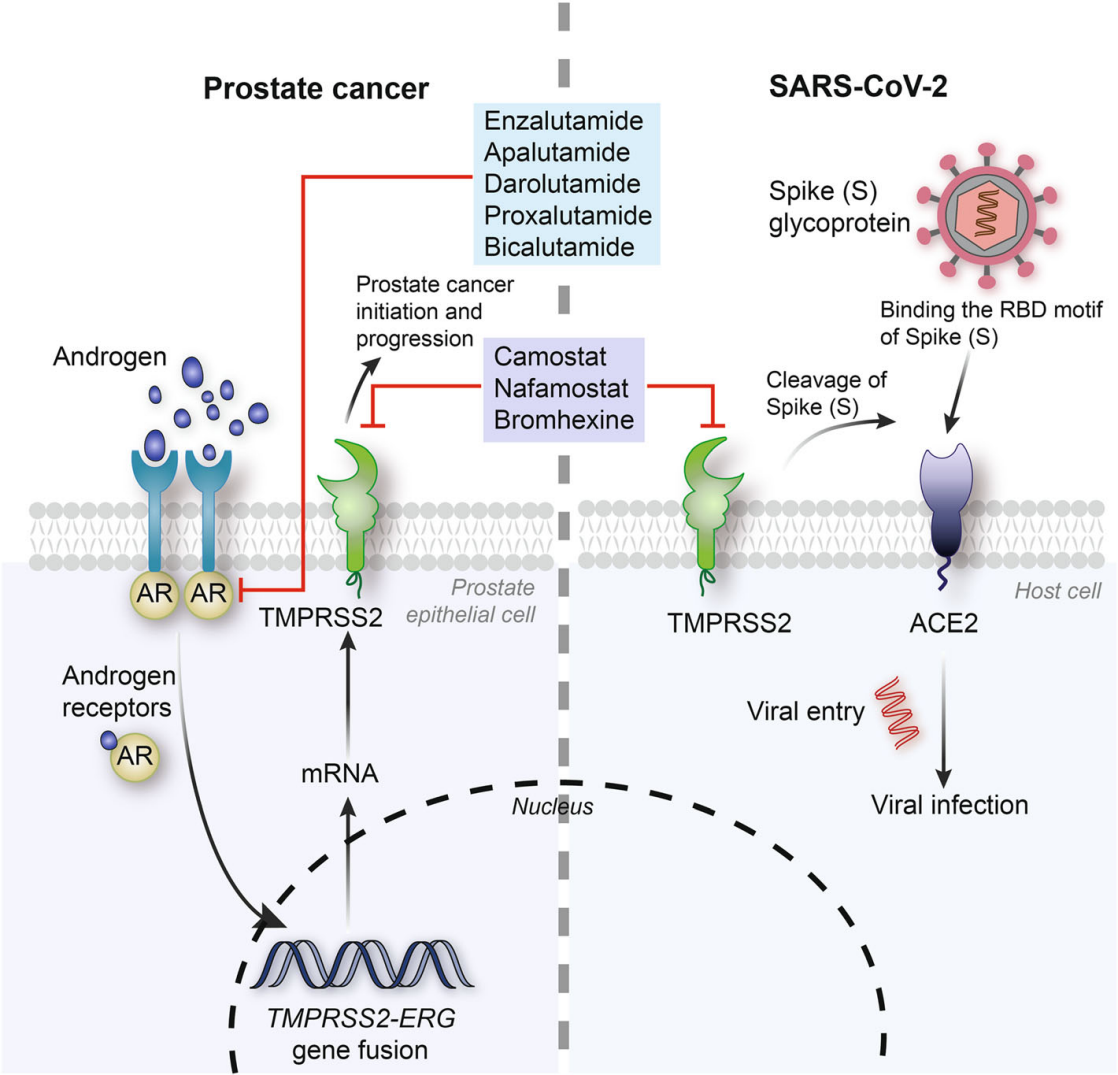
Androgen receptor (AR) signaling in prostate cancer and coronavirus disease 2019 (COVID-19). The AR is activated in response to androgens and induces the transcription of transmembrane serine protease 2 (*TMPRSS2*). The androgen-regulated TMPRSS2 gene may fuse with the erythroblast transformation-specific (ETS)-related gene (*ERG*), and this fusion is a molecular marker of prostate cancer initiation and progression. In severe acute respiratory syndrome coronavirus 2 (SARS-CoV-2) infection, TMPRSS2 is the protease that mediates S protein priming, which is essential for the interaction between SARS-CoV-2 and angiotensin-converting enzyme 2 (ACE2) and subsequent cell entry. Anti-androgen drugs and TMPRSS2 inhibitors used in patients with prostate cancer may serve as potential treatments for patients with COVID-19

ARs belong to the superfamily of hormonal nuclear receptors [162]. ARs are sequestered into the cytoplasm by heat shock proteins (HSPs) without ligand binding [163]. In response to androgens, ARs are activated and undergo a conformational change, leading to their nuclear translocation to initiate transcriptional activity by binding to androgen response elements (ARE) as a dimer [164–167]. One of the target genes of ARs is *TMPRSS2*, which encodes a type II transmembrane protein with serine protease activity [168, 169]. Administration of androgens leads to a profound increase in *TMPRSS2* expression, accompanied by androgen-dependent loading of the AR onto the *TMPRSS2* enhancer [170]. The TMPRSS2 protein is primarily expressed in the prostate secretory epithelium, and its expression level is highly upregulated in response to androgen signals [169]. Intriguingly, the androgen-regulated *TMPRSS2* gene may fuse with the erythroblast transformation-specific (ETS)-related gene *ERG*, the most common member of the oncogenic ETS family (Fig. 4). Notably, using a sequencing-based approach, a study demonstrated that the expression level of the fusion gene is regulated by DNA methylation patterns, providing a mechanism for tumor formation [171].

#### TMPRSS2 in prostate cancer

TMPRSS2 has been implicated in prostate cancer, not long after its discovery and cloning. The mRNA expression level of TMPRSS2 is robustly increased in prostate cancer in response to androgen stimulation [169]. The *TMPRSS2*-*ERG* gene fusion is well recognized as a molecular sign of prostate cancer, as it occurs in over half of primary prostate cancer cases [172, 173]. Compared to TMPRSS2-ERG fusion-negative prostate cancer, fusion-positive prostate cancer harbors distinct risk factors. For instance, higher genetically determined transcriptional activity of the AR in men is positively correlated with a higher risk of fusion-positive than fusion-negative prostate cancer [174]. Mouse experiments showed that prostate cancer metastasis is more likely to be promoted in the presence of TMPRSS2 [122]. Moreover, due to higher insulin/insulin-like growth factor signaling, men with prostate cancer harboring the TMPRSS2-ERG gene fusion are more susceptible to obesity and hence have poorer prognosis than those not harboring the gene fusion [175]. These findings suggest that differential *TMPRSS2* expression patterns may be a key determinant of prostate cancer risk.

#### TMPRSS2 in COVID-19

As outlined above, male patients with COVID-19 are prone to develop more severe complications and have worse clinical outcomes than females [176]. Moreover, low fatalities were observed in prepubertal children regarding SARS-CoV-2 infection [177]. In another study, patients with prostate cancer receiving androgen-deprivation therapy (ADT) were significantly less vulnerable to COVID-19 than those not treated with ADT [20]. These phenomena suggest that androgens play a role in COVID-19 severity and progression, and prostate cancer may be linked to COVID-19.

The spike (S) protein of coronavirus is composed of two components, S1 and S2. S1 is responsible for recognizing and binding of the virus to the host cell surface, while S2 is responsible for fusing the viral and cell membranes, allowing viral entry. TMPRSS2 is the protease that mediates S protein cleavage at the S1/S2 site, thus setting S1 and S2 apart. This event is called S protein priming and is believed to be essential for the interaction of SARS-CoV-2 with ACE2 and cell entry [2, 178]. ACE2 and TMPRSS2 are co-expressed in several cell types with a high susceptibility to SARS-CoV-2, such as type II pneumocytes, absorptive enterocytes of the small intestine, and nasal goblet secretory cells [179]. Prior to SARS-CoV-2 studies, TMPRSS2 was reported to be involved in H1N1 influenza virus infection. In comparison with wild-type mice, *Tmprss2* knockout mice avoided severe infection and thus escaped from lung diseases, highlighting the importance and conservation of TMPRSS2 function in viral entry events [180]. In a lung cancer tissue array, it was found that distinct types of lung cancer are androgen receptor-positive, indicating that in addition to prostate tissues, androgen signaling also targets the lung to enhance the expression of TMPRSS2 [170]. Considering the connection between androgens and *TMPRSS2* expression, especially in the lungs, the predominance in the number of COVID-19 deaths among males may be partially explained by high androgen levels and hence sustained *TMPRSS2* expression [20, 181]. However, similar TMPRSS2 expression levels in males and females were observed in lung tissues from mice and humans, which challenges the decisive role of TMPRSS2 in gender differences in COVID-19 outcomes [182]. Furthermore, it is worth noting that the disparities in infection risks do not sufficiently account for the gender-associated differential expression of *TMPRSS2*. To illustrate, *ACE2* is another gene that is highly expressed in males, especially in urogenital system organs, such as the prostate. A recent study demonstrated that patients with chronic urinary diseases are highly prone to SARS-CoV-2 infection [183].

#### Targeting TMPRSS2

Considering the indispensable function of TMPRSS2 in the pathogenicity of SARS-CoV-2, several therapeutics that are effective in attenuating androgen receptor signaling could be repurposed for the treatment of patients with COVID-19. Studies have revealed that administration of estrogen (like estradiol) or AR antagonists remarkably decreased *TMPRSS2* expression. Commercially available androgen antagonists include enzalutamide, apalutamide, darolutamide, and proxalutamide [184]. These anti-androgen drugs have been shown to be effective and safe for the treatment of prostate cancer for decades, and are promising for mitigating symptom severity in patients with SARS-CoV-2 by downregulating TMPRSS2 levels. ADT, the standard first-line therapy for androgen-sensitive prostate cancer, has already shown benefits in helping patients with prostate cancer stave off SARS-CoV-2 infections.

Apart from targeting TMPRSS2 expression, an alternative approach involves dampening TMPRSS2 protease activity. This is achieved using TMPRSS2 protease inhibitors, such as camostat, nafamostat, and bromhexine [122]. Among them, camostat has completed secondary outcome measures in clinical trials, and the entire study is estimated to be completed on May 1, 2021 (ClinicalTrials.gov; NCT04321096). Bromhexine, another potent TMPRSS2-specific protease inhibitor, was identified through large-scale chemical library screening and was demonstrated to lower the risk of metastasis in prostate cancer with no systemic toxicity shown [122].

Despite the availability and convenience of repurposing existing TMPRSS2-targeted drugs to suppress SARS-CoV-2, some issues need to be considered. First, modulating androgens appears to alter ACE2 expression. It was reported that in rat aorta, testosterone downregulated ACE2 mRNA and protein levels, while testosterone withdrawal showed the opposite effects [185]. Another study in rats revealed that chronic administration of the antiandrogen flutamide, without modulating estradiol levels, significantly boosted renal ACE2 mRNA expression [186]. Furthermore, transgender males showed higher levels of ACE2 expression and more cells expressing ACE2 after receiving ADT than normal individuals, as analyzed in microarray datasets [187]. Accordingly, the upregulated ACE2 expression following androgen suppression should be further investigated and counter-balanced to determine the potential impact on SARS-CoV-2 infection. Second, *TMPRSS2* mRNA and proteins are expressed not only in the prostate and lung but also in other tissues, such as the liver [188, 189]. A growing concern is that TMPRSS2-targeted therapy may result in unexpected outcomes in various tissues with normal physiological characteristics. TMPRSS2 activates the prostate-specific antigen via a proteolytic cascade in normal prostate tissues [122]. Nevertheless, others pointed out that the role of TMPRSS2 should not be overestimated, as its action may be compensated by that of other proteases, as evidenced by a study using *Tmprss2* knockout mice, where *Tmprss2* seemed dispensable for organ growth, development, and function [190].

In general, these findings unveil TMPRSS2 as a bond connecting prostate cancer and COVID-19, paving the way for repurposing conventional drugs that have few on-target side effects for treating COVID-19 based on androgen suppression and TMPRSS2 protease inhibition. The results of ongoing clinical trials are eagerly awaited.

### Immune checkpoint signaling

**Fig. 5:**
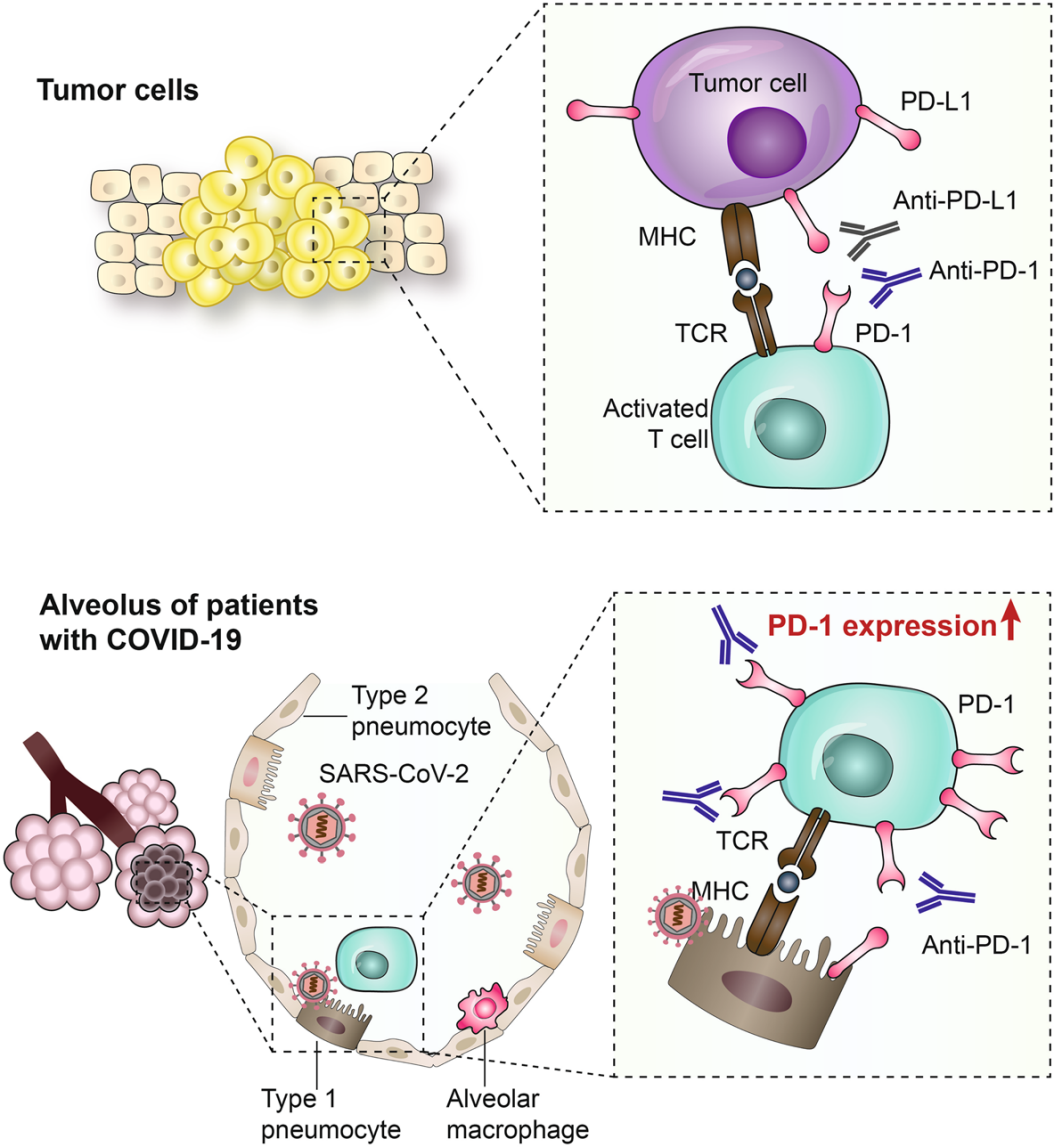
Immune checkpoint signaling in cancer and coronavirus disease 2019 (COVID-19). Immune checkpoint signaling involves several key steps. T cell receptors (TCRs) recognize their cognate antigens present on major histocompatibility complexes (MHCs) on antigen-presenting cells (APCs) or tumor cells. Several immune checkpoint molecules (such as programmed death (PD)-1) and their respective ligands (such as PD-L1) interact with each other to limit the hyperactivation and duration of the immune response. Immune checkpoint inhibition (ICI) treatment, such as anti-PD-1 antibodies, reactivates the cytotoxic T cell response against cancer and SARS-CoV-2 infection

Immune checkpoint signaling is an immunosuppressive pathway that involves the interaction of immune checkpoint molecules expressed on immune cells (especially T cells) with their corresponding ligands, thus responding to pathogens or malignant cells. Many specific checkpoint ligands are expressed on antigen-presenting cells (APCs) and other target cells [191, 192]. Immune checkpoint signaling involves several key steps. First, T cell receptors (TCRs) on antigen-specific T cells recognize their cognate antigens presented on the major histocompatibility complex (MHC) on APCs. Then, CD80/CD86 on APCs must provide signals to CD28 presented on T cells. Subsequently, several different immune checkpoint molecules (receptors) and their respective ligands interact with each other to limit the hyperactivation and duration of the immune responses [193] (Fig. 5). The most widely studied receptor-ligand combinations include programmed cell death protein 1 (PD1)-programmed cell death 1 ligand 1 (PDL1, also known as B7-H1) and cytotoxic T lymphocyte antigen 4 (CTLA4)-CD80/CD86. It is well established that checkpoint signals play a crucial role in maintaining immune tolerance and reducing autoimmunity, yet many pathogens and malignancies may utilize this pathway to escape immune surveillance by upregulating these checkpoint molecules.

#### Immune checkpoint inhibition

Given the upregulation of immune checkpoint molecules in tumor cells, immune checkpoint inhibition (ICI) has emerged as a promising therapeutic pillar in oncology, as discussed elsewhere [123–125].

In addition to cancer, several chronic and acute infectious diseases, such as malaria, human immunodeficiency virus (HIV) infection, and hepatitis B virus (HBV) infection, also exhibit high expression level of PD-1. Thus, ICI may contribute to viral clearance and has been evaluated in the treatment of infectious diseases [193, 194]. A clinical study showed acceptable ICI efficacy in 73 patients with cancer who contracted HIV, as over 90% of the subjects had suppressed viral load and increased CD4+ T cell count [126]. Moreover, ICI treatment was reported to be safe and efficient in patients with HBV/HCV infection and advanced-stage cancer, such as NSCLC [127, 128].

Despite the evidence supporting ICI effectiveness, ICI-based therapy appears more intricate in the context of chronic infectious diseases. For example, ICI is usually not readily adopted in patients with cancer having chronic infections, such as HIV [195]. This may be attributed to the compromised T cell function, which may reduce ICI efficacy or overproduction of inflammatory cytokines that cause organ injuries [196]. It is now suggested that patients with HIV having CD4+ T cell counts more than 350 cells/μL are suitable for ICI trials to boost ICI performance [197].

#### ICI in COVID-19

An urgent issue is whether there is an additional risk of ICI therapy for cancer patients during the COVID-19 pandemic. As mentioned above, owing to immunosuppression, patients with cancer receiving anti-cancer therapy, such as chemotherapy and radiotherapy, are highly prone to COVID-19 infection. Although some data showed that patients receiving ICI seemed to develop severe COVID-19 symptoms and show a high hospitalization rate [16], most of the clinical results support no relationship between the risk or severity of COVID-19 infection and ICI treatment in patients with cancer [25, 129, 198, 199]. To illustrate, a recent retrospective study of 1545 patients with cancer treated with ICIs showed no significant increase in the rate of COVID-19 after adjusting for demographics, medical comorbidities, and local infection rates [200].

Mounting evidence has shown the upregulation of immune checkpoint receptors in severe COVID-19 cases, which is associated with T cell exhaustion and lymphopenia [201–204]. In particular, after SARS-CoV-2 infection, cytokine storms induce T-cell hyperactivation and culminate in exhaustion, which is associated with concurrent lymphopenia [205]. In patients with severe COVID-19, CD8+ T cells reduce the cellular-mediated immune response to the virus and simultaneously upregulate immunosuppressive markers, such as PD-1 and mucin-3 [206, 207]. The disruption of T cells associated with severe COVID-19 may lead to viral sepsis and ARDS [208, 209]. As ICI targets immune checkpoint receptors and boosts both the number and function of cytotoxic T cells, the manifestations of COVID-19 render ICI a considerable option to treat COVID-19. Immunotherapies, such as convalescent plasma therapy, human monoclonal antibodies, and interferon, have proved to be safe and efficient in the treatment of COVID-19 [210]. To date, several clinical trials are underway to test the efficacy of ICIs for treating COVID-19, as listed in Table 1. Immune checkpoint receptors include PD1, as well as novel receptors, such as NKG2A and C5aR. Preclinical studies have revealed that inhibition of these immune checkpoint receptors strengthens T cell expansion and anti-tumor immunity. In particular, NKG2A inhibition enhances the anti-tumor performance of T cells and natural killer (NK) cells [211, 212].

Despite these advantages, some concerns remain regarding the application of ICI for treating COVID-19. ICI may reactivate exhausted T cells, forming immune competence. However, reinvigorated T cells may also augment cytokine secretion and increase the risk of the cytokine storm, ultimately leading to unfavorable organ injuries [213, 214]. Another concern involves ICI administration in patients with cancer and COVID-19. A meta-analysis study reported that patients with cancer treated with ICI have a risk of pneumonitis, namely, checkpoint inhibitor pneumonitis [215]. This may lead to a potential synergistic effect, as checkpoint inhibitor pneumonitis may exacerbate the poor symptoms of COVID-19-pneumonitis. Although checkpoint inhibitor pneumonitis as an adverse event is relatively rare, more investigations on the overlap between checkpoint inhibitor pneumonitis and COVID-19-pneumonitis are needed to better guide the administration of ICI in patients with both cancer and COVID-19.

## Conclusions

The widespread COVID-19 pandemic’s death toll is high among some portions of the population, including patients with cancer. In particular, cancer type, staging, and therapeutics affect the incidence and prevalence of SARS-CoV-2 infection. There are currently conflicting results regarding the mortality rates of patients with cancer who develop COVID-19. Further studies are required to determine whether cancer per se is an independent risk factor for developing COVID-19. During this special period, treating patients with both cancer and COVID-19 is considerably challenging, raising a desperate quest for the treatment that kills two birds with one stone. With joint efforts, we have accumulated an unprecedented perspective on the clinical relevance and molecular interactions governing the incidence and severity of both diseases. Given the close association between SARS-CoV-2 and cancer biology, cancer therapeutics capable of inhibiting SARS-CoV-2 infection and improving COVID-19 symptoms hold considerable promise to be repurposed as antivirals (Table 1).

Despite the benefits of repurposing, cancer therapeutics may aggravate the comorbidities of COVID-19. For example, increased hospitalization and severe respiratory conditions were reported to be side effects of treating COVID-19 with immune checkpoint inhibitors [16]. Treatment of patients with checkpoint inhibitor-based immunotherapy may stimulate the production of IL-6 and initiate the cytokine storm [216–218]. Importantly, administration of the IL-6R inhibitor tocilizumab has been proven to relieve cytokine release and enable patients to continue receiving ICI-based therapy [219, 220]. In addition, a number of studies have found that IL-6/JAK/STAT signaling induces the expression of PD-1 and/or PD-L1 [221–223]. Consequently, targeting IL-6/JAK/STAT signaling may lead to decreased ICI efficacy, as their targets, PD-1 and PD-L1, are downregulated. Combined targeting of IL-6 and PD-L1 resulted in enhanced inhibitory effects on tumor progression in mouse models of both pancreatic cancer and hepatocellular carcinoma [224, 225]. The JAK inhibitor ruxolitinib was shown to significantly improve the efficacy of immune checkpoint blockade therapy by impairing systemic inflammation in the tumor microenvironment and thus upregulating CTL infiltration and activation to overcome resistance to anti-PD-1 antibodies in pancreatic cancer [226]. Notably, a recent study suggested that other modulators of the innate immune system, such as TLR agonists or antagonists, may be implicated in anti-PD-1 treatment as an alternative [227]. Enlighted by the favorable combination of therapies in cancer treatments, future endeavors should be dedicated to developing combination approaches to treat COVID-19 to minimize the side effects of the existing monotherapies.

Another concern regarding COVID-19 treatment is the non-selectivity of JAK inhibitors. JAK inhibitors tend to suppress the activity of multiple cytokines. In addition, IL-6 and IFN-I share common downstream signaling molecules, including JAK1 and TYK2. This requires careful consideration of the administration of JAK inhibitors (or TYK2 inhibitors) in the context of inhibiting IL-6-mediated inflammatory responses, as the IFN-I responses are not supposed to be suppressed due to their positive roles in early infection.

As this formidable crisis continues to rage worldwide, it will be of vital significance to further investigate the clinical interactions of COVID-19 and cancer to better tailor the treatment of patients with cancer during the COVID-19 outbreak. The molecular interplay that involves the tight relationship between COVID-19 and cancer is not fully understood. Further studies are required to gain more insights into the biological mechanisms underlying the susceptibility and mortality of patients with cancer who develop COVID-19. Moreover, the molecular insights derived from basic research can be translated into clinical utility, which may expand the therapeutic approaches against COVID-19.

## Data Availability

Not applicable.

## Abbreviations

COVID-19: Novel coronavirus disease 2019
SARS-CoV-2: Severe acute respiratory syndrome coronavirus 2
IFN-I: Type I interferon
AR: Androgen receptor
S: Spike protein
ACE2: Angiotensin-converting enzyme 2
TMPRSS2: Transmembrane serine protease 2
ARDS: Adult respiratory distress syndrome
GDP: Gross domestic product
IL: Interleukin
TNF-α: Tumor necrosis factor α
CRS: Cytokine release syndrome
JAK: Janus kinase
STAT: Signal transducer and activator of transcription
IL-6R: IL-6 receptor
IL-6Rβ: IL-6 receptor subunit-beta
sIL-6R: soluble IL-6R
NSCLC: Non-small cell lung cancer
EMT: Epithelial-to-mesenchymal transition
CAR: Chimeric antigen receptor
BAL: Bronchoalveolar lavage
NF-κB: Nuclear factor-κB
XPO1: Exportin 1
SINE: Selective inhibitors of nuclear export
FDA: US Food and Drug Administration
BTK: Bruton tyrosine kinase
TLR: Toll-like receptor
CRP: C-reactive protein
ALL: Acute lymphoblastic leukemia
GVHD: Graft-versus-host disease
AAK1: AP2-associated protein kinase 1
IRF: Interferon regulatory factor
ISG: IFN-stimulated gene
PBMC: Peripheral blood mononuclear cell
HSP: Heat shock protein
ARE: Androgen response elements
ERG: Erythroblast transformation-specific-related gene
ADT: Androgen-deprivation therapy
APC: Antigen-presenting cell
TCR: T cell receptor
MHC: Major histocompatibility complex
PD1: Programmed cell death protein 1
PDL1: Programmed cell death 1 ligand 1
CTLA4: Cytotoxic T lymphocyte antigen 4
ICI: Immune checkpoint inhibition
HIV: Human immunodeficiency virus
HBV: Hepatitis B virus
NK: Natural killer
